# Privacy and contact tracing efficacy

**DOI:** 10.1098/rsif.2022.0369

**Published:** 2022-09-21

**Authors:** Sebastian Benthall, Erez Hatna, Joshua M. Epstein, Katherine J. Strandburg

**Affiliations:** ^1^ New York University, School of Law, 40 Washington Sq So, 10012 New York, NY, USA; ^2^ New York University, School of Global Public Health, 708 Broadway, New York, NY 10003, USA

**Keywords:** contact tracing, privacy, simulation

## Abstract

As the COVID-19 pandemic emerged, public health authorities and software designers considered the possibility that smartphones could be used for contact tracing to control disease spread. Smartphone-based contact tracing was attractive in part because it promised to allow the tracing of contacts that might not be reported using traditional contact tracing methods. Comprehensive contact tracing raises distinctive privacy concerns, however, that have not been previously explored. Contacts outside of an individual’s ordinary social network are more likely to be privacy-sensitive, making fear that such contacts will be disclosed a potential disincentive to adoption of smartphone contact tracing. Here, we modify the standard SEIR infectious disease transmission model to incorporate contact tracing and perform a series of simulations aimed at studying the importance of tracing socially distant (and potentially privacy-sensitive) contacts. We find that, for a simple model network, ensuring that distant contacts are traced is surprisingly unimportant as long as contact tracing adoption is sufficiently high. These results suggest that policy-makers designing contact tracing systems should be willing to trade off comprehensiveness for more widespread adoption.

## 1. Introduction

As the COVID-19 pandemic emerged, public health authorities and software designers considered the possibility that smartphones could be used for contact tracing—the practice of tracking the infection route of a contagious disease in order to monitor and control its spread [1]. Smartphones are attractive contact tracing devices because of their capacity to track locations, detect contacts, record information and provide two-way communication with public health authorities [2]. The hope was that smartphone contact tracing could improve on traditional interview-based contact tracing by providing real-time contact updating and notification and, significantly for the work reported here, allowing the tracing of contacts that might otherwise go unreported, such as contacts between strangers sharing a subway car.

Privacy is an important consideration in contact tracing policy, for reasons including stigma associated with some diseases [3,4], reluctance to disclose some sorts of relationships and contacts [5], and concern about government or industry reuse of personal information [6–8]. The idea of integrating smartphones into contact tracing heightened privacy concerns in various ways in comparison with traditional person-to-person contact tracing methods [9,10]. States and various private entities around the world raced to devise contact tracing solutions that would incorporate privacy-by-design and privacy regulation into smartphone-based contact tracing systems [11]. Ultimately, like other attempts at mobile contact tracing before them [12], these attempts at privacy-sensitive smartphone-based contact tracing applications became a small part of a larger failure to use contact tracing to curb COVID-19 in the USA [13,14]. The many roots of this failure included both public health officials’ resistance to software designs that minimized their access to information about disease spread [15] and individuals’ resistance to adopting the smartphone-based contact tracing apps that were deployed. Certain characteristics of COVID-19, such as its significant asymptomatic spread and aerosol transmission, also made contact tracing more difficult.

While varying in their implementation, the COVID-19 smartphone-based contact tracing initiatives have shared a relatively monolithic understanding of privacy that emphasizes individual control over (non-)disclosure of both COVID status and contact networks with little recognition of the different social implications of different sorts of disclosures. Recent surveys, on the other hand, have revealed the complexity of privacy preferences with respect to different classes of data and different cultural expectations [9].

These concerns about privacy and smartphone-based contact tracing transcend the COVID epidemic and are relevant to future outbreaks. The discussion to date is particularly insensitive to the likelihood that individuals will associate different levels of privacy concern with tracing of different sorts of social contacts. In particular, we posit that many people will be relatively unconcerned about revealing routine social contacts within a densely connected social group, since those contacts, such as those with family, friends and co-workers, are essentially in plain view. In other words, when a person is part of a densely connected social group, their contacts with others in the group are widely known. Tracing those contacts is relatively less likely to evoke privacy concerns.

When a person interacts with somebody who is not part of their densely connected social community, that connection is more likely to be unknown. Such unknown socially distant contacts may include connections such as those with mental health providers, abortion providers, political or activist groups, or illicit sexual partners that individuals would not want exposed to those in their densely connected social groups. Tracing these socially distant connections is thus more likely to evoke privacy concerns.

To illustrate the point, consider the following narratives:

— A person is a member of a large and close-knit family or social group. They have a liaison with somebody in a different group that would be socially unacceptable to members of their close-knit community. (It could be, for example, an extramarital affair, a non-heterosexual relationship, or a relationship outside of the ethnic, cultural or class expectations of the community.)
— A person is an employee of a company where co-workers frequently meet. They take time off of work, saying that it is for a sick day. But really they are interviewing for another job.
— A person is experiencing mental health issues and seeks psychological counselling or psychiatric care, but does not wish to share this information within their close-knit community.
— A person is involved in political activism or religious practices that they do not want to share with their co-workers or bosses.

Out-of-network contacts, which will tend to connect one densely connected social community with another, are likely to be particularly important for the spread of disease. Naively, one might anticipate that tracing such contacts is also critically important for preventing epidemic spread. Indeed, smartphone-based contact tracing is designed in part to catch contacts that might go unreported in traditional contact tracing, whether due to ignorance, faulty memory or intentional omission. This potential benefit to smartphone-based contact tracing may be undermined, however, if enough individuals decide not to install the software because of concerns about potential disclosure of privacy-sensitive contacts.

Whether an app-based contact-tracing program facilitates or blocks users from preventing the tracing of privacy-sensitive contacts is a design question. Here, we take a first step towards analysing this question by using a simple model to investigate the relative impact of tracing socially close and socially distant contacts. Within the model, and for reasons we deem likely to generalize, there is surprisingly little benefit to sacrificing app adoption in the pursuit of more thorough tracing of distant contacts. Depending on the extent to which concerns about privacy-sensitive contacts deter app adoption (an empirical question which is beyond the scope of this paper), it may be sensible to design smartphone contact tracing apps to provide wiggle room for individuals to keep some of their contacts ‘off the record'.

More generally, we view this work as a proof of concept for the value of using agent-based modelling in the design of contact tracing systems. Designing more effective contact tracing paradigms for future epidemics will require coordinated development of legal regulation and software systems, incorporating expert knowledge about both epidemiology and privacy. Epidemic spread, social information flow and technology adoption are complex systems that are likely to exhibit feedback effects, nonlinear dynamics and tipping points. Standard quantitative policy-making tools, such as traditional cost-benefit analysis are a poor fit for achieving regulatory goals in such systems [16–19].

This work builds on prior simulation studies evaluating the efficacy of smartphone-based contact tracing. Other studies have focused on the rate of smartphone contact tracing adoption needed to suppressed the pandemic, with estimates ranging from 32 to 60% of the total population [20–22] depending on modelling assumptions about the population, quarantining conditions and disease transmissibility. While other agent-based studies have focused on incorporating more realistic social network structures and imperfect testing efficiency [23], we have opted for a simplified model to focus on the effects of privacy concerns on contract tracing efficacy.

## 2. Methods

Public health has long used agent-based models of the transmission of contagions and behaviour between agents to design regulations to prolong and improve the quality of life [24–26]. Agent-based models of infectious diseases can augment traditional models based on dynamic equations with more realistic spatial and social dimensions, while also capturing nonlinearities and feedback effects that may elude such continuous approaches. In this work, we extend these modelling techniques to incorporate contact tracing’s impact on disease spread, allowing us to explore the privacy-related issues discussed above.

The simulation was built and analysed using scientific Python tools, including: NetworkX [27] and Seaborn [28].

### 2.1. Contact tracing model

Our model is a modification of the standard SEIR compartmental model. In the standard SEIR model, individuals are either *Susceptible (S)*, *Exposed (E)*, *Infectious (I)* or *Recovered (R)* (or Removed). In these models, *Exposed* individuals are infected with the pathogen. As explained in more detail below, *Susceptible* individuals have a chance of becoming *Exposed* if they come into contact with an *Infectious* individual. *Exposed* individuals become *Infectious* and eventually *Recovered* in a sequence governed by the model’s parameters. The standard SEIR model is characterized by the following dynamic equations:

$$\begin{array}{rl} & \frac{dS}{dt}=-\beta \frac{I}{N}S,\\ & \frac{dE}{dt}=\beta \frac{I}{N}S-\alpha E,\\ & \frac{dI}{dt}=\alpha E-\gamma I\\ \;\mathrm{and}\; & \frac{dR}{dt}=\gamma I, \end{array}$$

where *β* is the effective contact rate, *α* is the probability of an *Exposed* person becoming *Infectious* (equivalently, 1/*α* is the mean latent period), and *γ* is the probability of an *Infectious* person becoming *Recovered* (equivalently, 1/*γ* is the mean infectious period).

**Figure 1.**
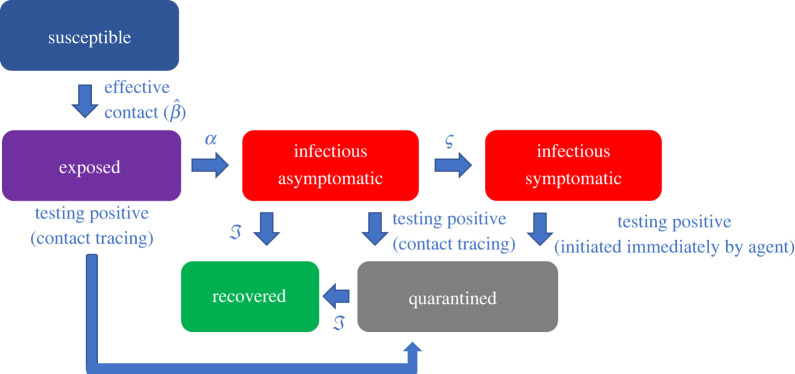
A flow chart of agent’s states.

Our model, illustrated in figure 1, is an agent-based computational modification of the SEIR model. It distinguishes between two types of *Infectious* individuals: *Asymptomatic Infectious* (*AI*) and *Symptomatic Infectious* (*SI*). After a period of latency, an *Exposed* individual becomes *AI*. That *AI* individual may (or may not) transition to the *SI* state later on. This scenario is consistent with common experience with COVID-19 and other contagious diseases.

We assume that *Recovered* individuals remain immune for the duration of the simulation and cannot be reinfected. Of course, as our experience with COVID-19 demonstrates, in a real epidemic it is possible for immunity to wane over time and reinfection to occur. Contact tracing is most likely to be useful and effective for suppressing an epidemic in the relatively early stages, however. Our assumption of no reinfection reflects our focus on such early stages and is consistent with the standard SEIR model.

To perform an agent-based simulation, we must model how contacts between individuals at each time step lead to the spread of disease. Here, we assume a graph of social connections that is fixed for the duration of the simulation. During each time step, a subset of these social connections are probabilistically activated as ‘contacts’ through which disease can potentially spread. The intuition behind this approach (which is obviously a simplification of real social interactions) is that social networks are relatively stable over the time periods relevant to our inquiry, but that individuals’ specific contacts within that network vary from day to day.

Specifically, the spatial and social connections between a set of *n* individuals, are represented using a weighted, undirected adjacency graph, in which each individual *i* is a node on the graph. An edge between two nodes indicates a non-zero probability that individuals *i* and *j* come into contact with each other at some point during the simulation. A *weight* *w*_*i*,*j*_ associated with the edge is the probability of contact during a single time step.

More specifically, adapting the classic SEIR model, we define parameters: $\hat{\beta }$, the probability of disease transmission from an *AI* or *SI* individual to a *Susceptible* individual given an active contact between them (thus turning the *Susceptible* individual *Exposed*); *α*, the probability that an *Exposed* person becomes *AI*; *ζ* (new to our model), the probability that an *AI* person becomes *SI*; and *γ*, the probability that an *AI or SI* person becomes *Recovered* (figure 1).

To model contact tracing, we assume that there is a testing mechanism that will always give a positive result for individuals who are *SI*, *Exposed* or *AI*.

In our simulations, we assume that *SI* individuals are tested at the moment the symptoms appear, while *Susceptible*, *Exposed* or *AI* individuals are tested only if they are identified through contact tracing. There are, of course, other approaches one might take to simulating contact tracing. For example, one could incorporate random testing of asymptomatic individuals (who might be *Susceptible*, *Exposed* or *AI*). Our approach is illustrative, but also, we think, consistent both with what happened early on in the COVID-19 pandemic and what is likely to occur early on in a future pandemic.

More specifically, the contact tracing process in our model begins when some individual becomes *SI* and, as a result, is tested. By assumption, that individual tests positive. The contact tracing system thus triggers a recursive wave of testing along traced contact paths. Any individual who tests positive is *Quarantined*. We assume that an individual who is *Quarantined* recovers during quarantine and is no longer susceptible to reinfection. This is consistent with our treatment of individuals who recover without being quarantined, as discussed above.

In the simulation, each individual is associated with a *contact list* *o*_*i*_ of other individuals who have been in contact with them at a previous time step, along with a time stamp. (One can imagine this list being stored in an individual’s smartphone or, equivalently, as a master list held by the contact tracing system.) To account for imperfections in the contact tracing technology, we define a *tracing probability* *c*_*i*,*j*_ for each edge, which is the probability that, if the edge is active, the contact will be added to the contact list of each of the individuals associated with that edge. When the contact tracing system is invoked, all contacts from the last *memory limit* *l* time steps are tested. In the simulations in this paper, we have fixed *l* = 10.

The simulation process proceeds as follows:

The simulation initializes the adjacency graph between individuals, assigning transmission weights *w*_*i*,*j*_ and contact tracing probabilities *c*_*i*,*j*_. All individuals begin as *Susceptible*, except for a small number of seed agents who are initialized as *AI*. At each time step *t*, a series of operations takes place over the simulated population:

1. For each edge (*i*, *j*), if neither *i* nor *j* is *Quarantined*, then the edge becomes active with probability *w*_*i*,*j*_, indicating that a contact between *i* and *j* takes place during that time step
2. For each active edge (*i*, *j*):
  (a) The contact becomes traceable with probability *c*_*i*,*j*_.
  (b) If *i* is *AI* and *j* is *Susceptible*, then *j* becomes *Exposed* with probability $\hat{\beta }$. And vice versa.
3. For each individual *i*:
  (a) If the individual is *Exposed*, they become *AI* with probability *α*.
  (b) If the individual is *AI* or *SI*, they become *Recovered* with probability *γ*
4. For each individual *i* who remains *AI*, with probability *ζ*:
  (a) individual *i* becomes *SI*
  (b) call subroutine: GetTested(*i*).

The subroutine, *GetTested*, can be called on an individual *i*. It calls itself recursively, simulating the contact tracing process. Once an individual is tested during a given time step, a flag, *T*_*i*_, is set to True.

When GetTested is called on an agent *i*:

1. If *T*_*i*_ is False:
  (a) Set *T*_*i*_ to True.
  (b) If the agent is *Exposed* or *AI* or *SI*:
    i. Set the agent to *Quarantined*
    ii. For all traceable contacts *j* of *i* up to *l* (the memory limit) time steps ago:
    iii. *GetTested*(*j*)

At the end of each time step, set *T*_*i*_ to false for all agents.

### 2.2. Network

**Figure 2.**
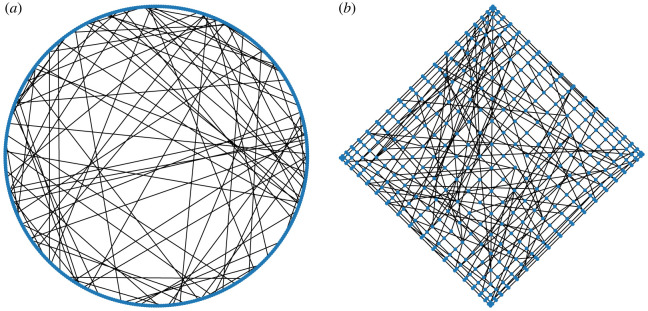
(*a*) A Watts–Strogatz ‘small world’ model. It is constructed first as one-dimensional lattice of nodes in which each node is connected to its *K* nearest neighbours using period boundary conditions. Then a proportion *p* of its edges are rewired to a random node. (*b*) A two-dimensional lattice with randomly rewired edges. In the original lattice, each nodes is connected to four neighbours. Though visualized here on a plane, the lattice has periodic boundary conditions and so is a torus. Here, we report results from our simulations using this two-dimensional extension of the Watts–Strogatz model.

We construct the network for our simulations with the goal of comparing the effects of tracing ‘close’ contacts within an individual’s densely connected local social environment with ‘distant’ contacts outside of those local communities. We build most directly on Lloyd *et al.* [29], who simulate the spread of infection on a small-world network, demonstrating that long-range contacts markedly increase the size and spread of the infection. We employ a two-dimensional generalization of the Watts–Strogatz model (figure 2). To create our simulated networks, we first construct a regular lattice (a two-dimensional torus) and then for each edge, with a fixed probability *p*, rewire one of its nodes to another, randomly chosen node. We define a ‘neighbourhood’ as a node’s four von Neumann neighbours in the original structure.

Our simulated networks thus have two kinds of edges: ‘short’ edges that were in a node’s neighbourhood in the original lattice structure, and ‘long’ rewired edges, which connect nodes that are not in the same neighbourhood. In turn we define ‘close’ contacts as those connected by short edges and ‘distant’ contacts as those connected by long edges. The proportion of contacts that are ‘distant’ is roughly equal to the rewiring probability *p* = 0.1. We take the more densely connected *close* contacts in an agent’s network to represent the widely observable in-community contacts discussed above, while long edges model the more privacy-sensitive out-of-community contacts. Assuming that individuals would, on average, be more willing to opt in to the tracing of *close* contacts than of *distant* contacts, we study how the efficacy of contact tracing depends on tracing those distant contacts. To measure efficacy, we monitor the proportion of the population that has been infected by the end of the simulation. We take a higher final infected proportion to indicate a worse epidemic. Contact tracing aims to reduce this final infected proportion.

To investigate the differential effects of tracing close and distant contacts, we define the contact tracing probability *c*_*i*,*j*_ for each edge as follows. We let *q* and *r* be the proportion of distant and close edges (respectively) that are traced (*c*_*i*,*j*_ = 1); for all untraced edges *c*_*i*,*j*_ = 0. By systematically varying *q* and *r*, we are able to compute the marginal effect of tracing close versus distant contacts on final infected proportion.

To produce the data analysed in this study, we used three values of *q* ∈ {0.05, 0.50, 0.95} and 19 values of *r* ∈ {0.00, 0.25, …, 0.95}. Because the number of short edges outnumbers the number of long edges nine-to-one (because *p* = 0.1), this implies that each increment of both *q* and *r* corresponds to 180.205 edges or 0.045 of the total number of edges. In the analysis section, we will compare the effects of tracing an additional increment, approximately 180, of close contacts to the effects of tracing an additional increment of distant contacts.

## 3. Results

**Figure 3.**
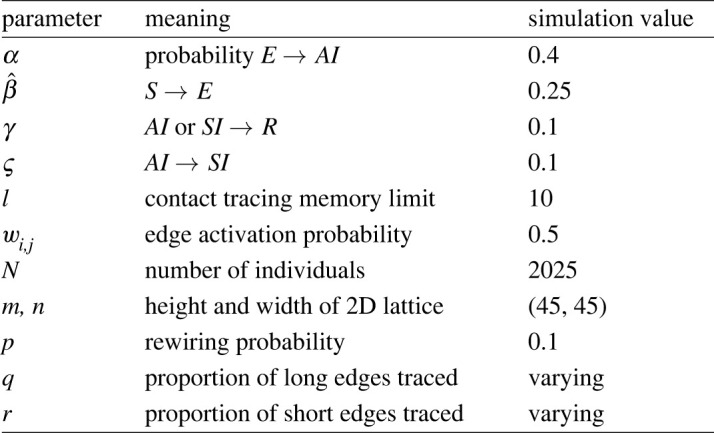
Parameter settings for the simulation.

We ran 1408 repetitions for each the 57 pairs of (*q*, *r*), resulting in 80 256 model runs. This design implies that the proportion of traced edges to all edges is a function of *q* and *r*: *T* = 0.1 *q* + 0.9 *r*. Moreover, (*q*, *r*) combinations of the form (0.05, *t*), (0.50, *t* − 0.05) and (0.95, *t* − 0.10), where *t* = *T* − 0.005, have the same corresponding *T* value. Figure 3 shows the parameter values used in these simulations.

**Figure 4.**
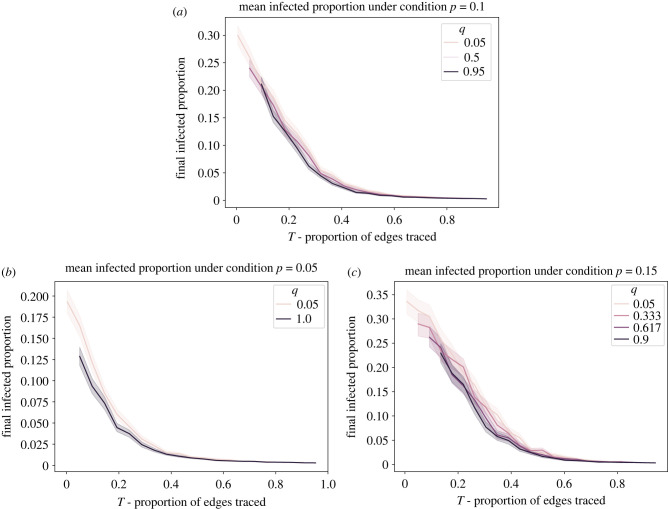
Mean of the final infected proportion as a function of the average total proportion of traced edges, *T*. Data are grouped by *q*, corresponding to the average ratio of distant traced edges to total traced edges in the simulation. The band around each line shows a standard deviation of the sample data above and below the mean value, or a 68% confidence interval. The main results reported in this paper are for *p* = 0.1 (*a*). For robustness, corresponding plots for *p* = 0.05 (*b*) and *p* = 0.15 (*c*) are included in this figure. (*a*) *p* = 0.10, (*b*) *p* = 0.05 and (*c*) *p* = 0.15.

Figure 4 shows the mean proportion of infected individuals. We see that the proportion decreases with the increase of the proportion of traced edges (*T*), Thus, as expected, tracing more contacts (whether close or distant) is generally more effective at suppressing infection spread. Moreover, figure 4 also illustrates that trading off between trading close and distant contacts for a given value of total traced contacts *T* has a relatively minor effect on the final infected proportion. Recall that for any given *T*, the level of *q* is traded off with *r* such that the difference between curves with different values of *q* is whether increments of 0.045 of the traced edges are long or short. For values of *T* above approximately 0.5 in our simulation, there is essentially no difference between the results with different *q* values, indicating that it makes little, if any difference, whether one traces close or distant contacts. Below approximately 0.5, the curves appear to diverge somewhat, with higher values of *q* corresponding to somewhat lower final infected proportions, meaning that all else equal, tracing distant contacts is slightly more effective in this range. Even below *T* = 0.5, the additional suppression of average final infected proportion due to tracing distant contacts is quite small compared with the effects of changing the overall number of contacts that are traced.

**Figure 5.**
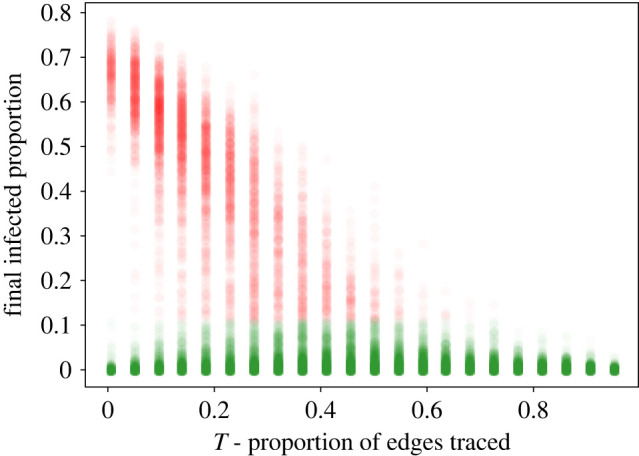
Scatter plot of final infected proportion as a function of *T* for each case in the study. In cases with low contact tracing rates, the distribution of the final infected proportion is bimodal. The cut-off rate of 0.11 is the highest value in the lower mode in the least traced condition. Below this rate, in green, the disease undergoes early extinction. Above this rate, we consider the disease to have become an epidemic.

However, looking only at the average of a large number of simulation runs conceals some potentially policy-relevant underlying differences in system behaviour. Figure 5 shows a scatter plot of the distribution of final infected proportion values for all runs at all values of *q* plotted as a function of *T*. When *T* is relatively high (above approximately 0.5 in our model), epidemic spread, in which a large proportion of the population is infected, essentially never occurs, though the amount of local spread continues to decrease at higher *T*.

In low contact tracing conditions (below approximately *T* = 0.5 in this model), while the average infected proportion rises gradually as contact tracing is scaled back, the distribution of the proportion of infected individuals becomes markedly bimodal. The average over many simulation runs with different random dynamics thus can mask the potential for substantial epidemic spread. Because real life does not give do-overs, we expect that policy-makers will be concerned not only with lowering the average final infected proportion, but also with reducing the chance of epidemic spread. We thus study the effects of tracing close and distant contacts on the occurrence of epidemic spread.

**Figure 6.**
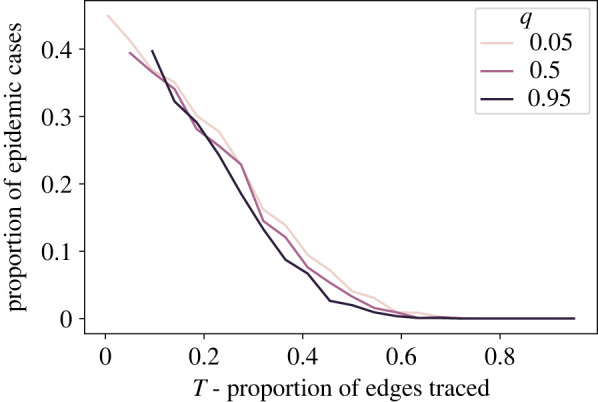
Proportion of cases with infected proportion over 0.11 as a function of *T*, grouped by *q*. Cases with a lower infected proportion are considered to have early extinction of the disease.

To measure the probability of epidemic spread, we define a cut-off infected proportion of 0.11 based on the highest value observed in the lower mode of the data in the least traced condition (0.05, 0.0). Cases with final infected proportion below this cut-off are counted as early extinctions without epidemic spread. Cases with final infected proportion above this cut-off are counted as epidemics. This cut-off relative to the lower mode is illustrated in figure 5. Given these definitions, we can calculate the proportion of epidemics observed at each of our parameter values. (This cut-off definition is somewhat arbitrary, but does not significantly affect our qualitative observations.) The results are plotted in figure 6.

For levels of *T* > 0.6, whether or not the traced contacts are close or distant has little effect on the proportion of simulations that become epidemics; at these values of *T* almost no epidemics occur for any value of *q*. For lower values of *T*, however, tracing distant contacts appears to decrease the proportion of cases that become epidemics. At *T* = 0.4, for example, a change of 180 traced short edges (0.045 of the total number of edges in the network) into traced long edges may decrease the proportion of epidemics by 0.02.

## 4. Limitations

The study in this paper uses a static network structure with a stochastic chance of a network edge being active on each time step. A more general model would account for changes in network structure over time. We consider this limitation in light of the duration of epidemics given our model parametrization. Under the condition with minimal contact tracing, epidemics (cases with infected proportion over the 0.11 threshold) had a duration of mean 281.9 and standard deviation 54.1 time steps. Under the interpretation that a time step is a day, most of the outbreaks in our simulation are cleared in less than a year. A static contact network may be a realistic modelling choice in this case.

We have used a modified (Watts–Strogatz) small-world network because it is well known and offers a clear distinction between close (local) links and rewired links (which represent far-away contacts in the context of our investigation). However, we have considered all agents connected by rewired edges to be ‘distant’ without regard to Euclidean distance between them in the original lattice. Future work can address different network topologies and operationalize edge sensitivity using finer-grained metrics.

## 5. Discussion

Previous studies have evaluated the cost and effectiveness of different levels of screening and contact tracing [30,31]. This study is motivated by questions raised by the roll-out of smartphone-based contact tracing in the COVID-19 pandemic. Discussions of smartphone contact tracing design have implicitly assumed that tracing such socially distant contacts is important for suppressing disease. This assumption is reasonable at first blush in light of studies such as [29], which find that long-range contacts are associated with larger infection proportions. We point out, however, that tracing socially ‘distant’ contacts is likely to be more socially costly in terms of privacy and of the effects of privacy concerns on people’s willingness to adopt contact tracing technology than tracing contacts between socially ‘close’ contacts—that is, people who are in a more locally clustered collection of nodes. Tracing more ‘distant’ contacts is also a logistical challenge for traditional contact tracing methods. As a result, we posit that there may be a trade-off between system design efforts aimed at ensuring that distant contacts are traced and efforts to increase the total amount of contact tracing that occurs.

Given the likelihood of such a trade-off, we use a simple agent-based model to explore whether and to what extent tracing distant contacts is important to contact tracing efficacy. We find that, when contact tracing of close contacts is sufficiently robust, there is minimal to no advantage to expending effort and resources on tracing distant contacts.

When contact tracing is ‘leakier’, in that fewer contacts are traced and many potentially infectious contacts slip through the cracks, the situation is a bit different, in that sometimes increasing the tracing of distant contacts can mean the difference between epidemic extinction and epidemic spread. However, even in such leaky contact tracing scenarios, the relative advantage of tracing distant contacts is quite small, raising the question for policy-makers and system-designers about the appropriate trade-off between investing in more comprehensive local tracing versus ensuring that distant contacts are traced.

Of course, our simulation results have no quantitative significance for policy-makers, based as they are on a very simplified representation of social interactions. We nonetheless believe that these results are qualitatively meaningful and can be understood in terms of the underlying mechanisms of infectious spread. The important insight is this: distant contacts are important for the spread of infection from one community to another because without them the disease can spread, if at all, only through long chains of close contacts, which offer many opportunities for failure to infect, slowing or even stopping the spread. Stopping spread by testing and quarantining does not depend as heavily on distant contacts, because once the disease spreads from one community to another, symptomatic members of the second community become independent sources for contact tracing. As long as those symptomatic individuals are identified quickly enough and there is robust tracing of their close contacts, epidemic spread in the second community will be suppressed. This perspective also helps us to understand why tracing distant contacts takes on somewhat more significance in situations where contact tracing is generally leakier.

## Data Availability

The software code and data used in this study have been archived on Zenodo under a CC-BY 4.0 license (doi:10.5281/zenodo.7011327) [32]. They have also been uploaded as part of the electronic supplementary material [33].
